# Supplementary figures and images for: Barcoded Asaia bacteria enable mosquito in vivo screens and identify novel systemic insecticides and inhibitors of malaria transmission
Source: PLoS Biol. 2021 Dec 20;19(12):e3001426. doi: 10.1371/journal.pbio.3001426 (PMC8726507; doi:10.1371/journal.pbio.3001426)

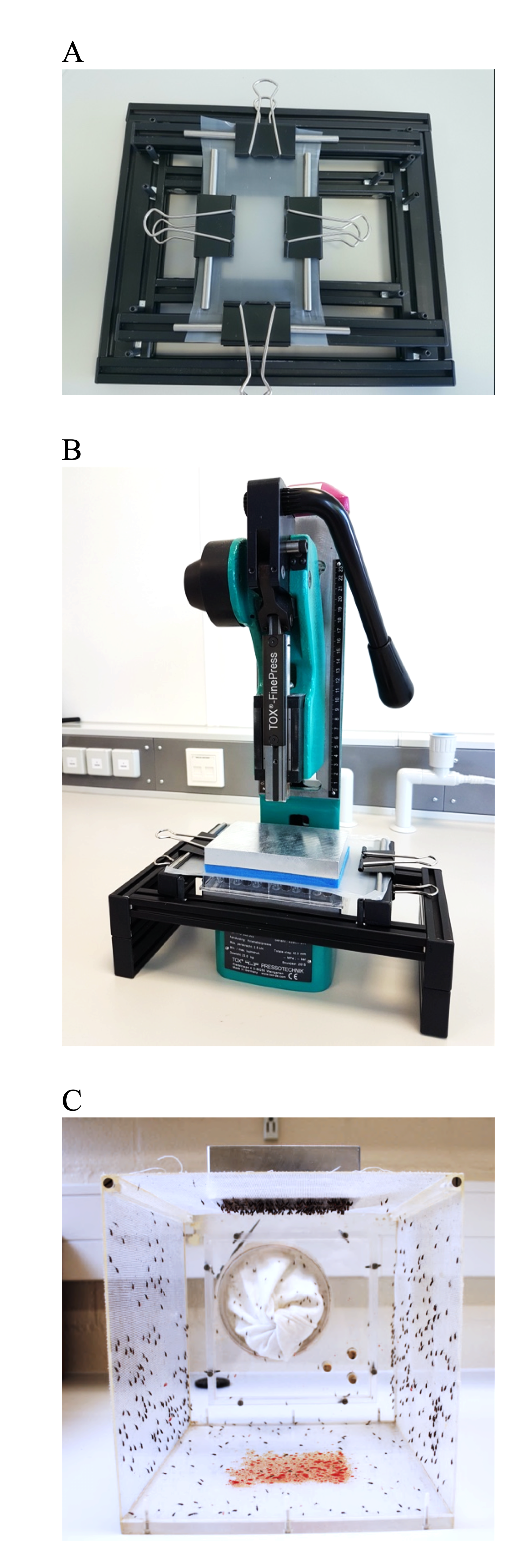

Supplement: S1 Fig — (A) Device for stretching Parafilm in 2 directions. (B) Lever press used for applying the Parafilm membrane to a 96-well plate. (C) Feeding mosquitoes on a 96-well plate. The plate is heated by an aluminium heat block on top of the plate. (TIFF) [file pbio.3001426.s002.tiff]

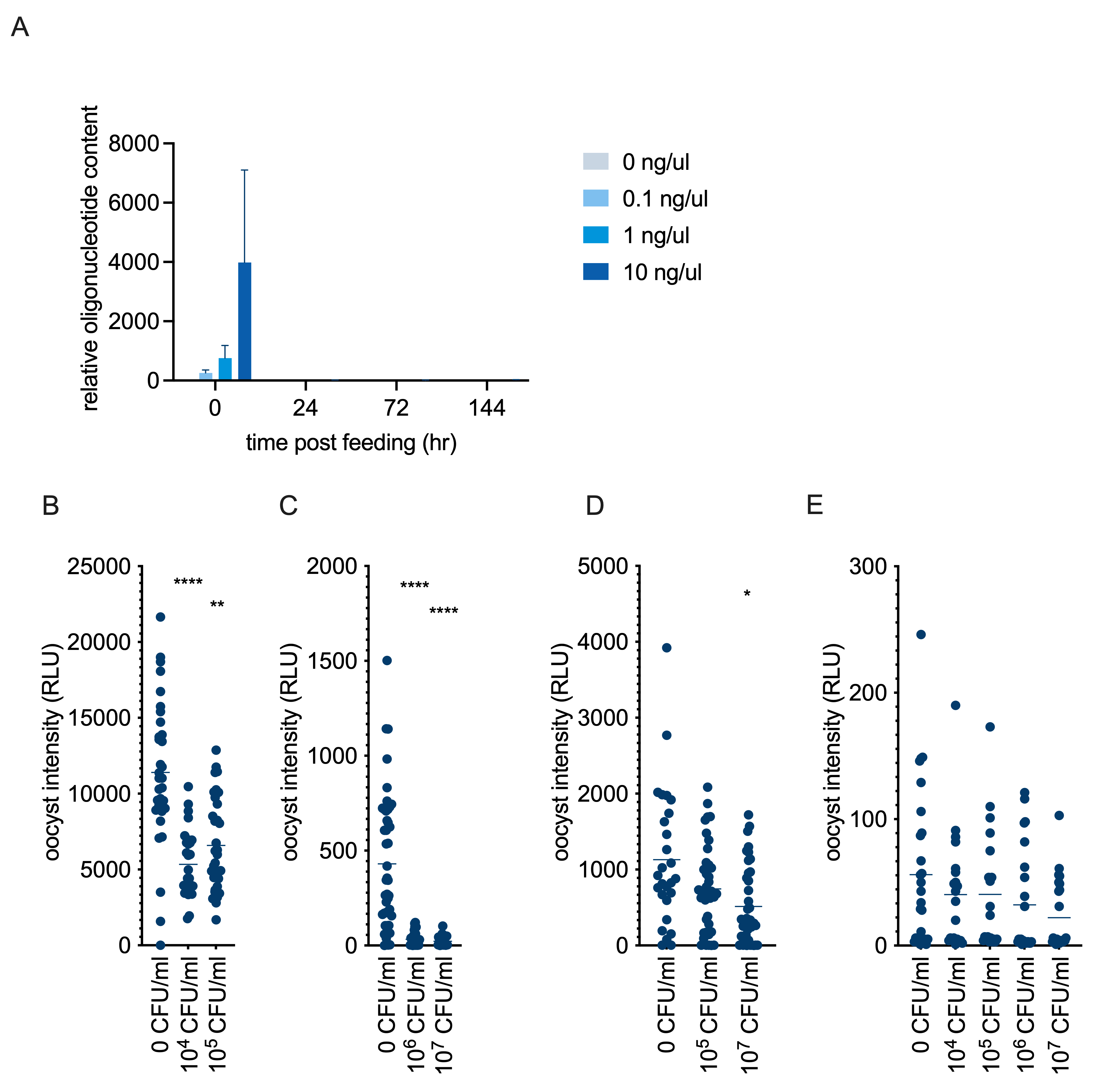

Supplement: S2 Fig — (A) Pilot experiment with blood meals tagged with a phosphorothioate oligonucleotide. Mosquitoes were fed by membrane feeding on blood meals containing 0 to 10 ng/μl of oligonucleotide as indicated in the legend. At 0, 24, 72, and 144 hours postfeeding, mosquitoes were homogenised, total DNA was isolated, and the amount of oligonucleotide was determined by semiquantitative real time PCR. (B–E) Effect of Pantoea agglomerans (B and C) or Asaia SF2.1 (D and E) on transmission of P. falciparum NF54 parasites. The panels show data from independent experiments. Experiments were conducted with parasite strain NF54-HGL that expresses a GFP-luciferase reporter throughout the life cycle. Stage V gametocytes were combined with bacteria at the densities indicated on the x-axis and fed to Anopheles stephensi mosquitoes. Eight days after feeding, infection status was determined by luminescence analysis. The symbols indicate relative light units observed in individual mosquitoes. Asterisks indicate data significantly different from the control infection that received no bacteria (*P < 0.05, **P < 0.01;****P < 0.0001). Underlying data for this figure can be found in S1 Data. (TIFF) [file pbio.3001426.s003.tiff]

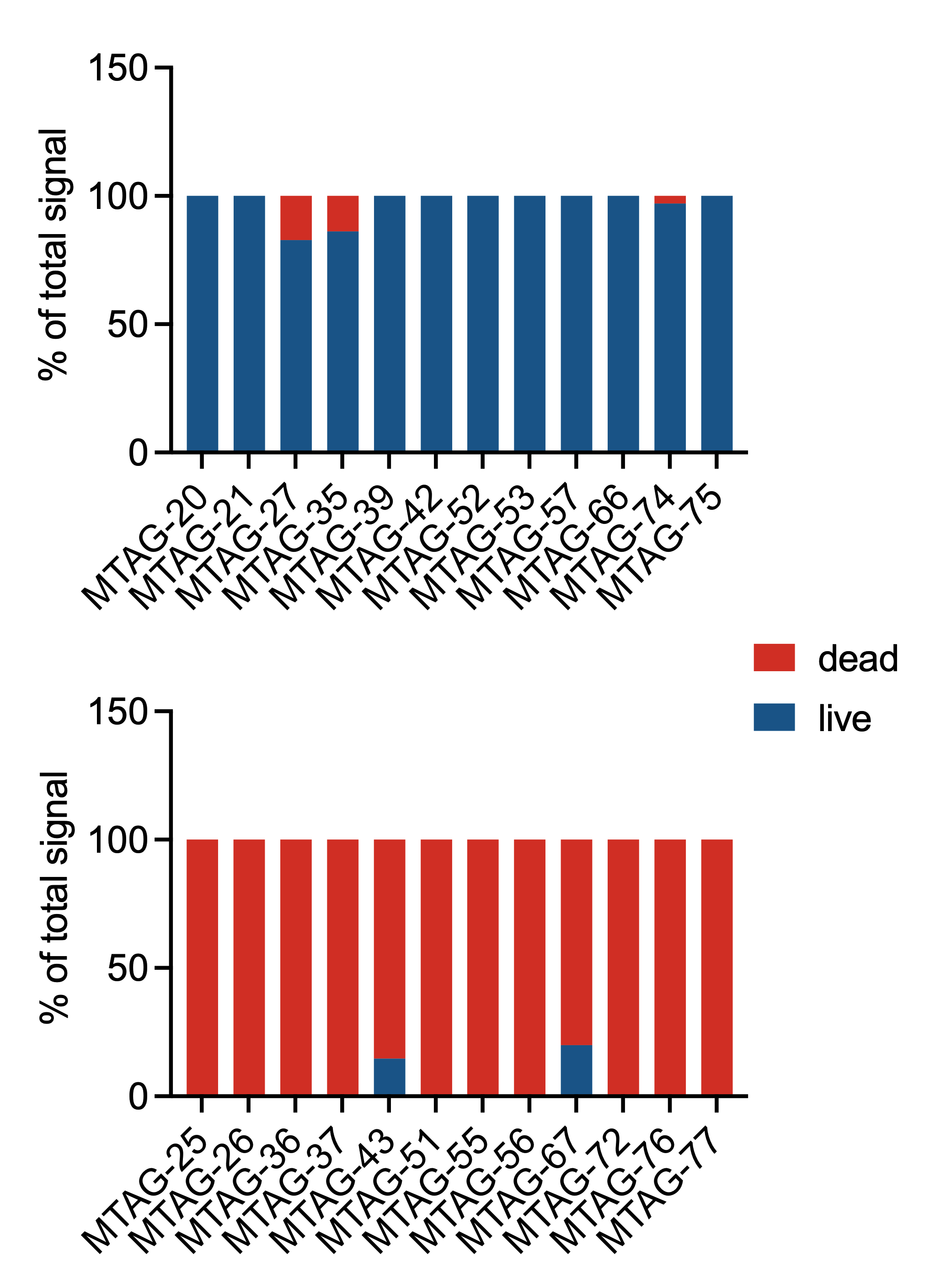

Supplement: S3 Fig — Barcode signals were quantified from individual mosquitoes. For each barcode, all signals were summed. The figure shows the percentage of signal that was derived from dead versus live mosquitoes. Underlying data for this figure can be found in S1 Data. (TIFF) [file pbio.3001426.s004.tiff]

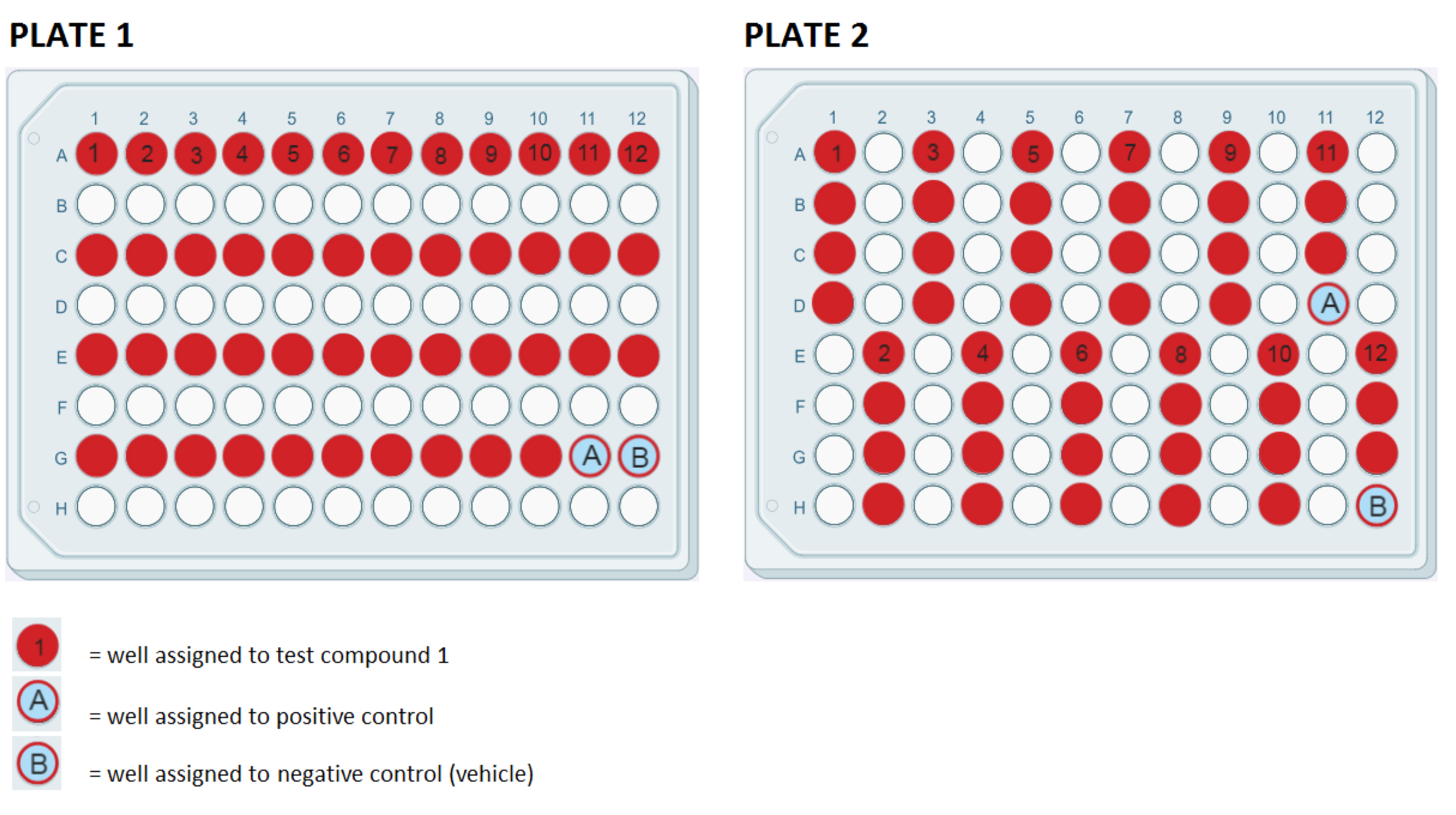

Supplement: S4 Fig — All samples were tested in duplicate plates using the plate maps indicated in the figure. (TIFF) [file pbio.3001426.s005.tiff]

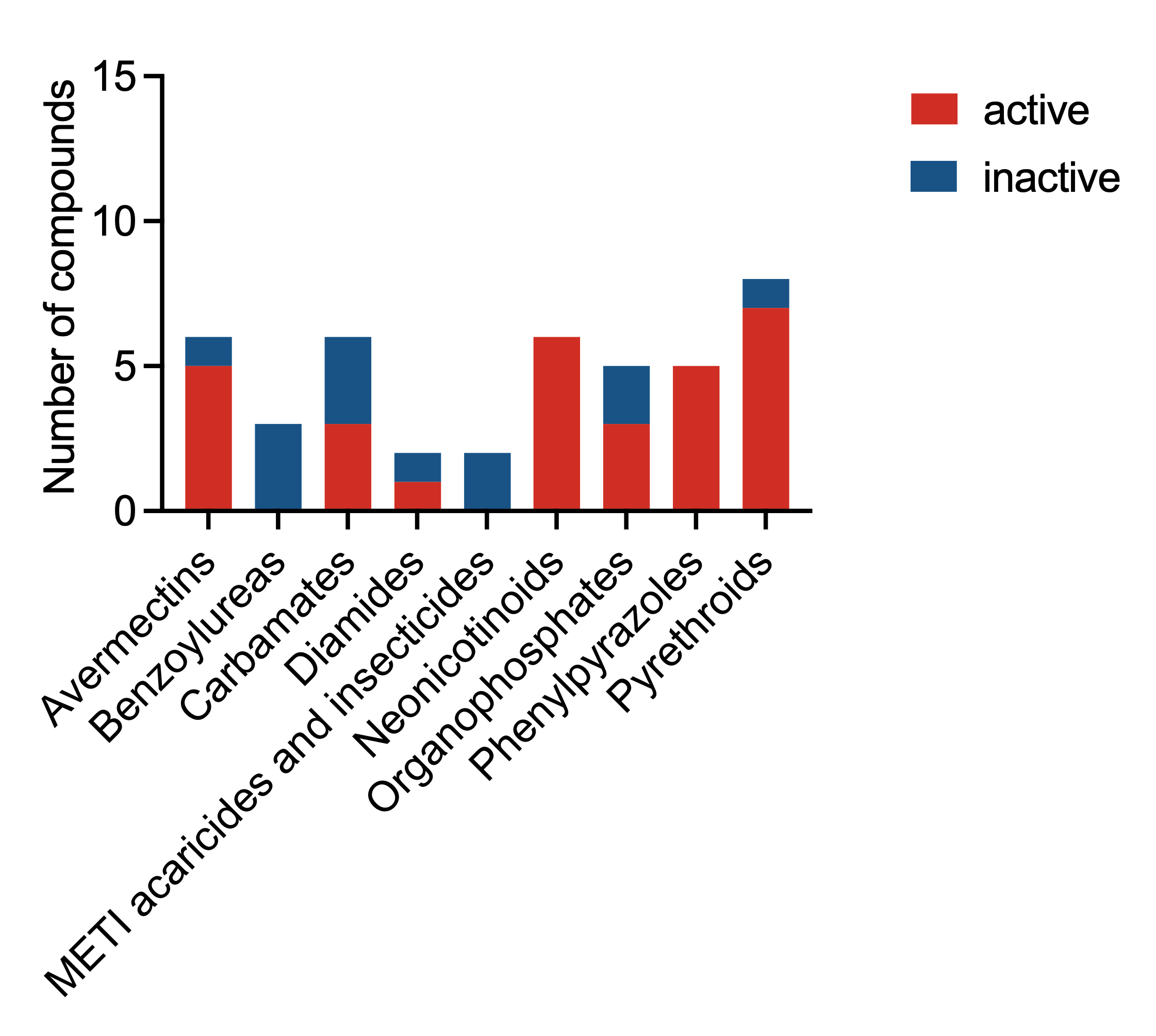

Supplement: S5 Fig — Compounds were considered active when the associated barcode showed ≥50% enrichment in dead mosquitoes at one of the test concentrations (0.1; 1.0; 10.0 μM). Unclassified compounds or classes composed of <2 compounds are not included in the figure. Underlying data for this figure can be found in S1 Data. (TIFF) [file pbio.3001426.s006.tiff]

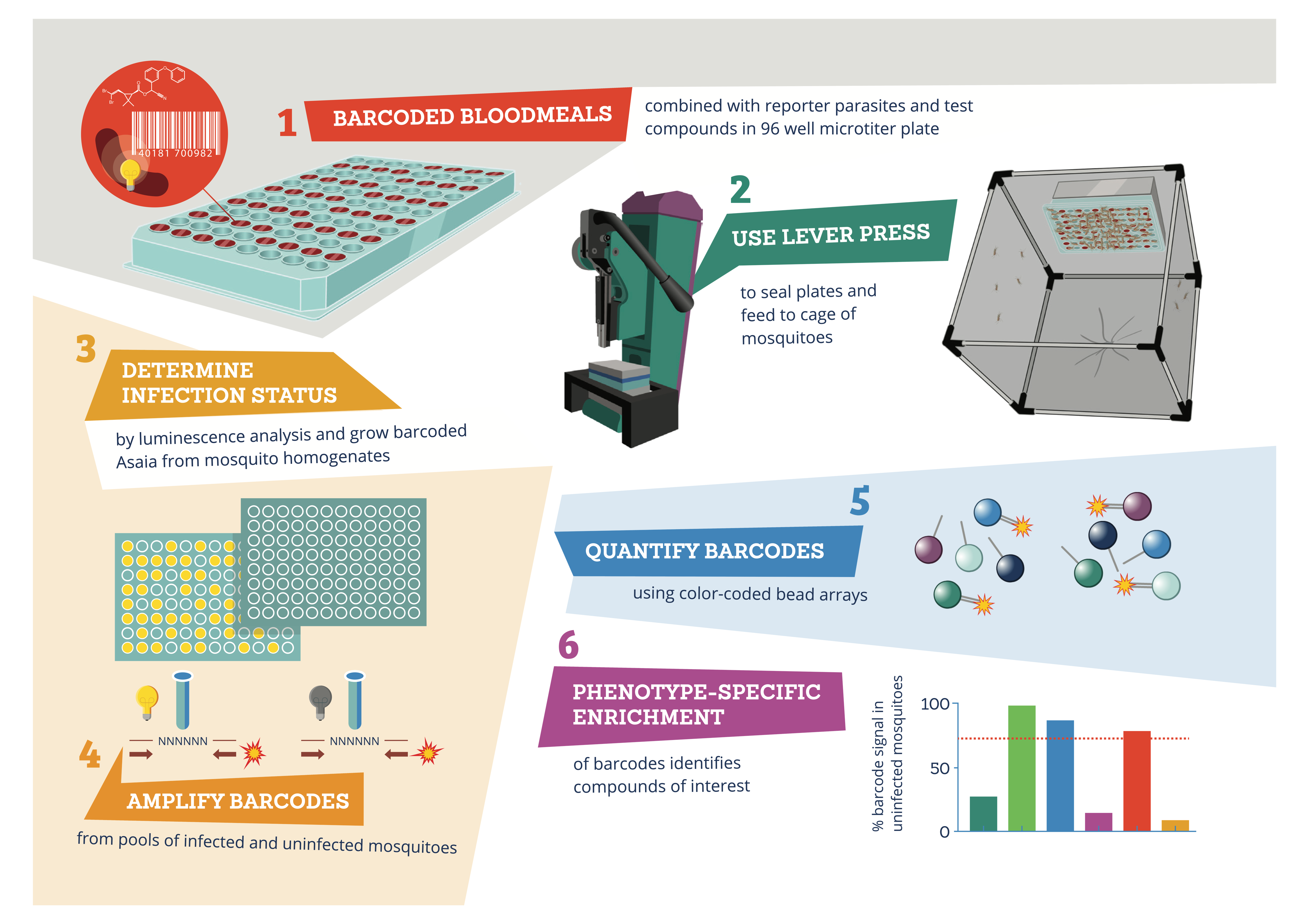

Supplement: S6 Fig — Test compounds were combined with infectious stage V gametocytes from Plasmodium falciparum strain NF54-HGL that expresses a luciferase reporter throughout the life cycle. Following 24-hour incubation, gametocytes were supplemented with uninfected red blood cells and barcoded Asaia bacteria and fed to Anopheles stephensi mosquitoes using 96-well microtiter plates. Eight days after blood feeding, infection status of individual mosquitoes was determined through luminescence assays. In parallel, Asaia bacteria were grown from homogenates of individual mosquitoes in 96-well liquid cultures under kanamycin selection pressure. Asaia were pooled according to infection status into pools for infected versus uninfected mosquitoes. Barcodes were then amplified by PCR using a fluorescently labelled primer pair that binds a common sequence flanking the DNA barcode sequence. Following amplification, barcodes were quantified by multianalyte profiling using DNA oligos coupled to colour-coded microspheres, which resulted in a fluorescence signal for each barcode depending on the quantity of the barcode in the PCR amplification product. Barcodes enriched in the uninfected mosquitoes identified compounds with malaria transmission-blocking activity, whereas detection of barcode signals from the infected mosquitoes were used to verify sampling of barcodes that were missing in the pool of uninfected mosquitoes. (TIFF) [file pbio.3001426.s007.tiff]

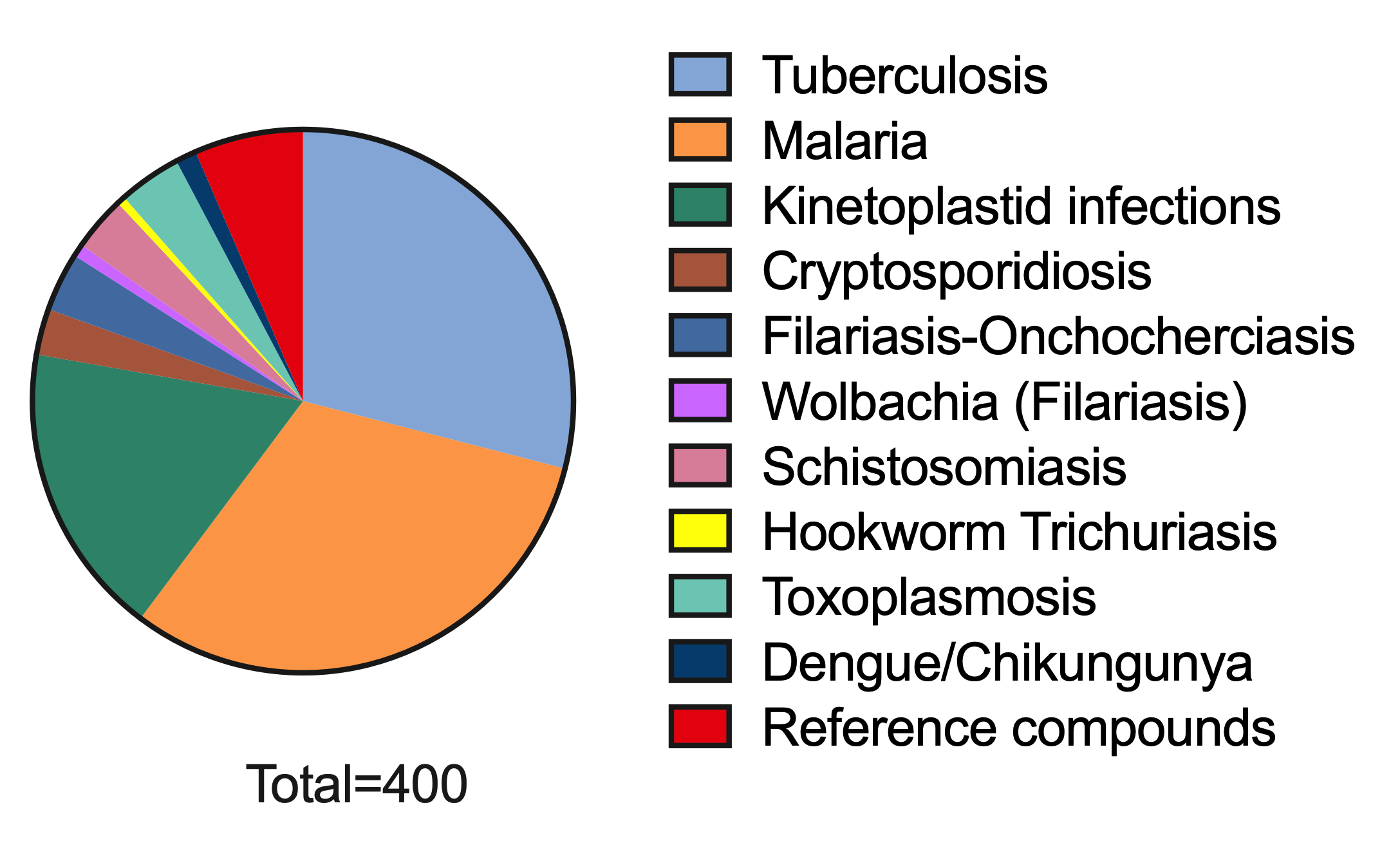

Supplement: S7 Fig — Underlying data for this figure can be found in S1 Data. MMV, Medicines for Malaria Venture. (TIFF) [file pbio.3001426.s008.tiff]

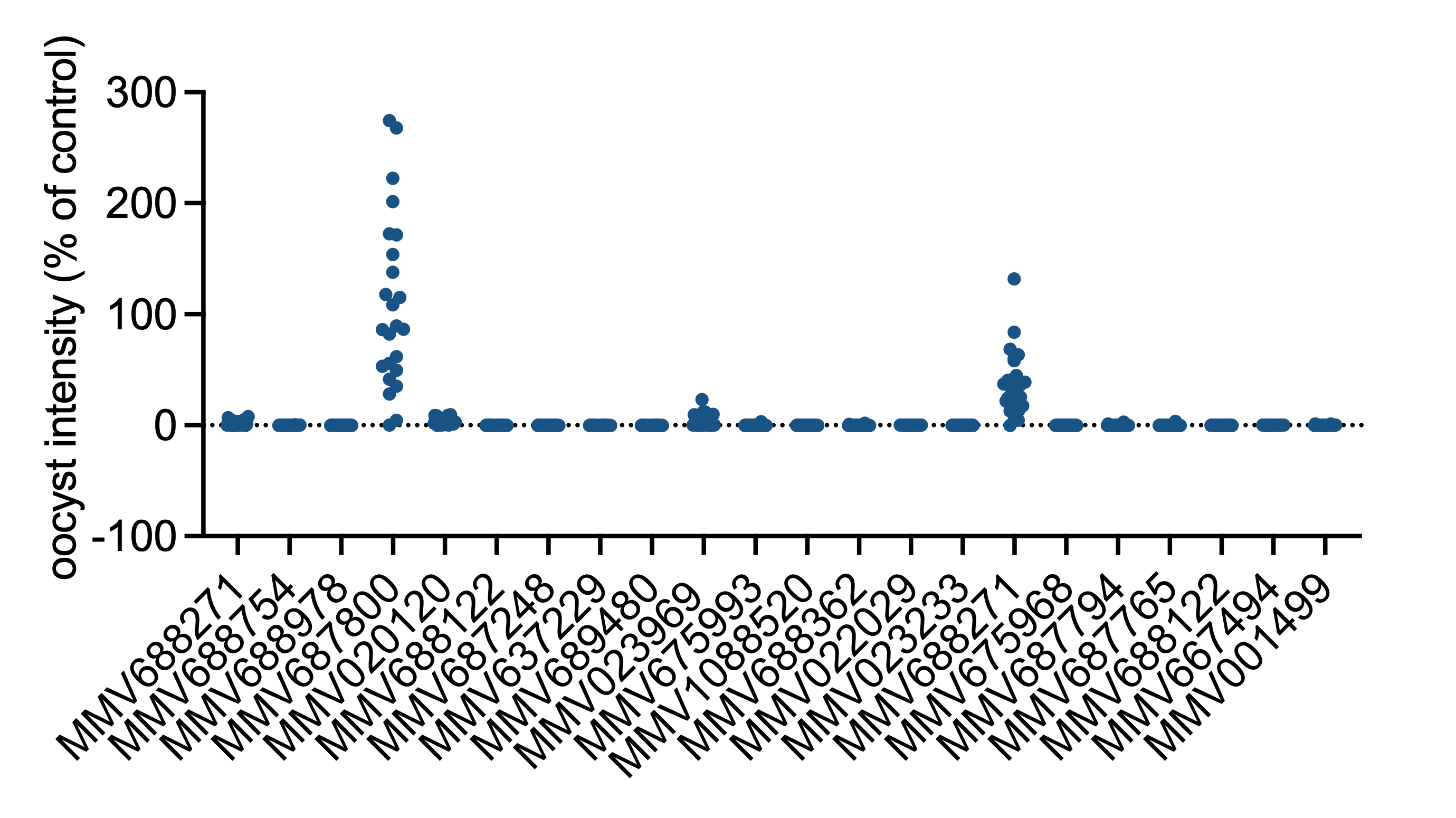

Supplement: S8 Fig — Experiments were conducted with P. falciparum strain NF54-HGL that expresses a luciferase reporter throughout the life cycle. Stage V gametocytes were preincubated with test compound for 24 hours prior to feeding to Anopheles stephensi mosquitoes. Eight days after feeding, infection status was determined by luminescence analysis. The symbols indicate relative oocyst intensities normalised to the luminescence signals observed in vehicle control (0.1% DMSO) infections. In line with the test concentrations in the barcoded screen, compounds MMV688754, MMV688978, MMV688122, MMV687248, MMV023969, MMV675993, MMV1088520, MMV688362, MMV022029, MMV687794, MMV687765, and MMV688122 were tested at 20 μM, all other compounds were tested at 10 μM. Underlying data for this figure can be found in S1 Data. MMV, Medicines for Malaria Venture. (TIFF) [file pbio.3001426.s009.tiff]
