# Supplementary material for: Barcoded Asaia bacteria enable mosquito in vivo screens and identify novel systemic insecticides and inhibitors of malaria transmission
Source: PLoS Biol. 2021 Dec 20;19(12):e3001426. doi: 10.1371/journal.pbio.3001426 (PMC8726507; doi:10.1371/journal.pbio.3001426)
Supplement: S1 Table — Underlined nucleotides indicate phosphorothioate modifications. (DOCX) [file pbio.3001426.s010.docx]

| **ID** | **Sequence** |
| --- | --- |
| Phosphorothioated sense sequence | 5’-GCTCTACGACAGCTGCCTACAACTGACTAACAATTGGAGGGCAAGATACAGATCGTGAGTGCTGACAGTAACTG |
| Phosphorothioated antisense sequence | 5’-CAGTTACTGTCAGCACTCACGATCTGTATCTTGCCCTCCAATTGTTAGTCAGTTGTAGGC AGCTGTCGTAGAGC |
| MWV 303 | 5’-CGACAGCTGCCTACAACTG |
| MWV 304 | 5’-CTGTCAGCACTCACGATCTG |
| 18s Pf-MGB Probe | 5’-FAM-AACAATTGGAGGGCAAG-NFQ |
| MWV 371 | 5’-TAACCATGGCTAGACTGTGACGAGTACTTGGACTAGTACAACATACGAGCCGGAAGC-3’ |
| MWV 374 | 5’GATGACGTCTAATACCAGTCAATCGACATGCACGCCTTAAGCTTCGAAGGTAACTGGCTTCAGC-3’ |
| MWV 358 | 5’-Biotin-TACCAGTCAATCGACATGCACGC-3’ |
| MWV 486 | 5’-CCTTGCGTATAATATTTGCCCATG-3’ |

Table S1. Oligonucleotides used in pilot barcoding experiments and PCR amplifications. Underlined nucleotides indicate phosphorothioate modifications.
