# Supplementary material for: Barcoded Asaia bacteria enable mosquito in vivo screens and identify novel systemic insecticides and inhibitors of malaria transmission
Source: PLoS Biol. 2021 Dec 20;19(12):e3001426. doi: 10.1371/journal.pbio.3001426 (PMC8726507; doi:10.1371/journal.pbio.3001426)
Supplement: S2 Table — (DOCX) [file pbio.3001426.s011.docx]

| **Primer ID** | **Sequence** |
| --- | --- |
| MWV 363 | CTAGTAGTAGAAAGTTGAAATTGATTATGGAATTCC |
| MWV 385 | TTAAGGAATTCCATAATCAATTTCAACTTTCTACTA |
| MWV 365 | CTAGTAGTGAATGTAAGATTATGTATTTGGAATTCC |
| MWV 386 | TTAAGGAATTCCAAATACATAATCTTACATTCACTA |
| MWV 367 | CTAGTATTGTGAAAGAAAGAGAAGAAATTGAATTCC |
| MWV 387 | TTAAGGAATTCAATTTCTTCTCTTTCTTTCACAATA |
| MWV 369 | CTAGTGTTGTAAATTGTAGTAAAGAAGTAGAATTCC |
| MWV 388 | TTAAGGAATTCTACTTCTTTACTACAATTTACAACA |
| MWV 393 | CTAGTGTAATTGAATTGAAAGATAAGTGTGAATTCC |
| MWV 394 | TTAAGGAATTCACACTTATCTTTCAATTCAATTACA |
| MWV 395 | CTAGTGTGTGTTATTTGTTTGTAAAGTATGAATTCC |
| MWV 396 | TTAAGGAATTCATACTTTACAAACAAATAACACACA |
| MWV 397 | CTAGTAAATTAGTTGAAAGTATGAGAAAGGAATTCC |
| MWV 398 | TTAAGGAATTCCTTTCTCATACTTTCAACTAATTTA |
| MWV 399 | CTAGTATTAAGTAAGAATTGAGAGTTTGAGAATTCC |
| MWV 400 | TTAAGGAATTCTCAAACTCTCAATTCTTACTTAATA |
| MWV 401 | CTAGTGATTGATATTTGAATGTTTGTTTGGAATTCC |
| MWV 402 | TTAAGGAATTCCAAACAAACATTCAAATATCAATCA |
| MWV 403 | CTAGTGTATGTTGTAATGTATTAAGAAAGGAATTCC |
| MWV 404 | TTAAGGAATTCCTTTCTTAATACATTACAACATACA |
| MWV 405 | CTAGTTTTGATTTAAGAGTGTTGAATGTAGAATTCC |
| MWV 406 | TTAAGGAATTCTACATTCAACACTCTTAAATCAAAA |
| MWV 407 | CTAGTAAGATGATAGTTAAGTGTAAGTTAGAATTCC |
| MWV 408 | TTAAGGAATTCTAACTTACACTTAACTATCATCTTA |
| MWV 409 | CTAGTGATAGATTTAGAATGAATTAAGTGGAATTCC |
| MWV 410 | TTAAGGAATTCCACTTAATTCATTCTAAATCTATCA |
| MWV 411 | CTAGTTTTAAGTGAGTTATAGAAGTAGTAGAATTCC |
| MWV 412 | TTAAGGAATTCTACTACTTCTATAACTCACTTAAAA |
| MWV 413 | CTAGTGTGTTATAGAAGTTAAATGTTAAGGAATTCC |
| MWV 414 | TTAAGGAATTCCTTAACATTTAACTTCTATAACACA |
| MWV 415 | CTAGTTATTAGAGTTTGAGAATAAGTAGTGAATTCC |
| MWV 416 | TTAAGGAATTCACTACTTATTCTCAAACTCTAATAA |
| MWV 417 | CTAGTTGATATAGTAGTGAAGAAATAAGTGAATTCC |
| MWV 418 | TTAAGGAATTCACTTATTTCTTCACTACTATATCAA |
| MWV 419 | CTAGTAATAAGAGAATTGATATGAAGATGGAATTCC |
| MWV 420 | TTAAGGAATTCCATCTTCATATCAATTCTCTTATTA |
| MWV 421 | CTAGTTTGTGTAGTTAAGAGTTGTTTAATGAATTCC |
| MWV 422 | TTAAGGAATTCATTAAACAACTCTTAACTACACAAA |
| MWV 423 | CTAGTTGTATATGTTAATGAGATGTTGTAGAATTCC |
| MWV 424 | TTAAGGAATTCTACAACATCTCATTAACATATACAA |
| MWV 425 | CTAGTAGTAAGTGTTAGATAGTATTGAATGAATTCC |
| MWV 426 | TTAAGGAATTCATTCAATACTATCTAACACTTACTA |
| MWV 427 | CTAGTTTGTGATAGTAGTTAGATATTTGTGAATTCC |
| MWV 428 | TTAAGGAATTCACAAATATCTAACTACTATCACAAA |
| MWV 429 | CTAGTATTTGTTATGATAAATGTGTAGTGGAATTCC |
| MWV 430 | TTAAGGAATTCCACTACACATTTATCATAACAAATA |
| MWV 431 | CTAGTAAATAAGAATAGAGAGAGAAAGTTGAATTCC |
| MWV 432 | TTAAGGAATTCAACTTTCTCTCTCTATTCTTATTTA |
| MWV 433 | CTAGTAATGTAAAGTAAAGAAAGTGATGAGAATTCC |
| MWV 434 | TTAAGGAATTCTCATCACTTTCTTTACTTTACATTA |
| MWV 435 | CTAGTGTTAGTTATGATGAATATTGTGTAGAATTCC |
| MWV 436 | TTAAGGAATTCTACACAATATTCATCATAACTAACA |
| MWV 437 | CTAGTGTGATTGAATAGTAGATTGTTTAAGAATTCC |
| MWV 438 | TTAAGGAATTCTTAAACAATCTACTATTCAATCACA |
| MWV 439 | CTAGTTATTGTTGAATGTGTTTAAAGAGAGAATTCC |
| MWV 440 | TTAAGGAATTCTCTCTTTAAACACATTCAACAATAA |
| MWV 445 | CTAGTTATGAATGTTATTGTGTGTTGATTGAATTCC |
| MWV 446 | TTAAGGAATTCAATCAACACACAATAACATTCATAA |
| MWV 447 | CTAGTGATAAGAAAGTGAAATGTAAATTGGAATTCC |
| MWV 448 | TTAAGGAATTCCAATTTACATTTCACTTTCTTATCA |
| MWV 449 | CTAGTGTAAGATTAGAAGTTAATGAAGAAGAATTCC |
| MWV 450 | TTAAGGAATTCTTCTTCATTAACTTCTAATCTTACA |
| MWV 451 | CTAGTGTTTGTGTTTGTATAAGTTGTTAAGAATTCC |
| MWV 452 | TTAAGGAATTCTTAACAACTTATACAAACACAAACA |
| MWV 453 | CTAGTTAGAGAAAGAGAGAATTGTATTAAGAATTCC |
| MWV 454 | TTAAGGAATTCTTAATACAATTCTCTCTTTCTCTAA |
| MWV 455 | CTAGTGAAGATATTGAAAGAATTTGATGTGAATTCC |
| MWV 456 | TTAAGGAATTCACATCAAATTCTTTCAATATCTTCA |
| MWV 457 | CTAGTAATTAGAAGTAAGTAGAGTTTAAGGAATTCC |
| MWV 458 | TTAAGGAATTCCTTAAACTCTACTTACTTCTAATTA |
| MWV 459 | CTAGTAGAGTATTAGTAGTTATTGTAAGTGAATTCC |
| MWV 460 | TTAAGGAATTCACTTACAATAACTACTAATACTCTA |
| MWV 461 | CTAGTTATTAGAGAGAAATTGTAGAGATTGAATTCC |
| MWV 462 | TTAAGGAATTCAATCTCTACAATTTCTCTCTAATAA |
| MWV 463 | CTAGTTGAAATGTGTATTTGTATGTTTAGGAATTCC |
| MWV 464 | TTAAGGAATTCCTAAACATACAAATACACATTTCAA |
| MWV 465 | CTAGTTTTGTTGTTAAGTATGTGATTTAGGAATTCC |
| MWV 466 | TTAAGGAATTCCTAAATCACATACTTAACAACAAAA |
| MWV 467 | CTAGTATGATGTGTTTGATTTGAATTGAAGAATTCC |
| MWV 468 | TTAAGGAATTCTTCAATTCAAATCAAACACATCATA |
| MWV 469 | CTAGTTGAGTAAGTTTGTATGTTTAAGTAGAATTCC |
| MWV 470 | TTAAGGAATTCTACTTAAACATACAAACTTACTCAA |
| MWV 471 | CTAGTGTAAGAGTATTGAAATTAGTAAGAGAATTCC |
| MWV 472 | TTAAGGAATTCTCTTACTAATTTCAATACTCTTACA |
| MWV 473 | CTAGTTTTGTGTGTTATTGTAATTGAGATGAATTCC |
| MWV 474 | TTAAGGAATTCATCTCAATTACAATAACACACAAAA |
| MWV 475 | CTAGTAATTGAGAAAGAGATAAATGATAGGAATTCC |
| MWV 476 | TTAAGGAATTCCTATCATTTATCTCTTTCTCAATTA |
| MWV 477 | CTAGTGTTGAGAATTAGAATTTGATAAAGGAATTCC |
| MWV 478 | TTAAGGAATTCCTTTATCAAATTCTAATTCTCAACA |
| MWV 479 | CTAGTAATGAAATAGTGTTAAATGAGTGTGAATTCC |
| MWV 480 | TTAAGGAATTCACACTCATTTAACACTATTTCATTA |
| MWV 481 | CTAGTTTTGTTAGAATGAGAAGATTTATGGAATTCC |
| MWV 482 | TTAAGGAATTCCATAAATCTTCTCATTCTAACAAAA |
| MWV 483 | CTAGTAAAGAATTAGTATGATAGATGAGAGAATTCC |
| MWV 484 | TTAAGGAATTCTCTCATCTATCATACTAATTCTTTA |
| MWV 506 | CTAGTGTTATGATATAGTGAGTTGTTATTGAATTCC |
| MWV 507 | TTAAGGAATTCAATAACAACTCACTATATCATAACA |
| MWV 508 | CTAGTGTATAGTGTGATTAGATTTGTAAAGAATTCC |
| MWV 509 | TTAAGGAATTCTTTACAAATCTAATCACACTATACA |

Table S2. Oligonucleotides used for generation of barcode plasmids.
