# Supplementary material for: Barcoded Asaia bacteria enable mosquito in vivo screens and identify novel systemic insecticides and inhibitors of malaria transmission
Source: PLoS Biol. 2021 Dec 20;19(12):e3001426. doi: 10.1371/journal.pbio.3001426 (PMC8726507; doi:10.1371/journal.pbio.3001426)
Supplement: S3 Table — (DOCX) [file pbio.3001426.s012.docx]

| **TAG region** | **hybridized primer pair** | **barcode construct ID** |
| --- | --- | --- |
| MTAG-012 | MWV 363/385 | MV172 |
| MTAG-013 | MWV 365/386 | MV173 |
| MTAG-014 | MWV 367/387 | MV174 |
| MTAG-015 | MWV 369/387 | MV175 |
| MTAG-018 | MWV 393/394 | MV176 |
| MTAG-019 | MWV 395/396 | MV177 |
| MTAG-020 | MWV 397/398 | MV178 |
| MTAG-021 | MWV 399/400 | MV179 |
| MTAG-022 | MWV 401/402 | MV180 |
| MTAG-025 | MWV 403/404 | MV181 |
| MTAG-026 | MWV 405/406 | MV182 |
| MTAG-027 | MWV 407/408 | MV183 |
| MTAG-028 | MWV 409/410 | MV184 |
| MTAG-029 | MWV 411/412 | MV185 |
| MTAG-030 | MWV 413/414 | MV186 |
| MTAG-033 | MWV 415/416 | MV187 |
| MTAG-034 | MWV 417/418 | MV188 |
| MTAG-035 | MWV 419/420 | MV189 |
| MTAG-036 | MWV 421/422 | MV190 |
| MTAG-037 | MWV 423/424 | MV191 |
| MTAG-038 | MWV 425/426 | MV192 |
| MTAG-039 | MWV 427/428 | MV193 |
| MTAG-042 | MWV 429/430 | MV194 |
| MTAG-043 | MWV 431/432 | MV195 |
| MTAG-044 | MWV 433/434 | MV196 |
| MTAG-045 | MWV 435/436 | MV197 |
| MTAG-046 | MWV 437/438 | MV198 |
| MTAG-047 | MWV 439/440 | MV199 |
| MTAG-048 | MWV 445/446 | MV200 |
| MTAG-051 | MWV 447/448 | MV201 |
| MTAG-052 | MWV 449/450 | MV202 |
| MTAG-053 | MWV 451/452 | MV203 |
| MTAG-054 | MWV 453/454 | MV204 |
| MTAG-055 | MWV 455/456 | MV205 |
| MTAG-056 | MWV 457/458 | MV206 |
| MTAG-057 | MWV 459/460 | MV207 |
| MTAG-061 | MWV 461/462 | MV208 |
| MTAG-062 | MWV 463/464 | MV209 |
| MTAG-063 | MWV 465/466 | MV210 |
| MTAG-064 | MWV 467/468 | MV211 |
| MTAG-065 | MWV 469/470 | MV212 |
| MTAG-066 | MWV 471/472 | MV213 |
| MTAG-067 | MWV 473/474 | MV214 |
| MTAG-072 | MWV 475/476 | MV215 |
| MTAG-073 | MWV 477/478 | MV216 |
| MTAG-074 | MWV 479/480 | MV217 |
| MTAG-075 | MWV 481/482 | MV218 |
| MTAG-076 | MWV 483/484 | MV219 |
| MTAG-077 | MWV 506/507 | MV226 |
| MTAG-078 | MWV 508/509 | MV227 |

Table S3. List of TAG regions on Luminex microspheres, primers used for barcode construction and resulting barcoded plasmids.
