# Supplementary material for: Barcoded Asaia bacteria enable mosquito in vivo screens and identify novel systemic insecticides and inhibitors of malaria transmission
Source: PLoS Biol. 2021 Dec 20;19(12):e3001426. doi: 10.1371/journal.pbio.3001426 (PMC8726507; doi:10.1371/journal.pbio.3001426)
Supplement: S4 Table — Plasmodium asexual blood stage and gametocyte inhibition data were retrieved from https://www.mmv.org/mmv-open/pathogen-box. SMFA, standard membrane feeding assay. (DOCX) [file pbio.3001426.s013.docx]

| Compound | Pathogen box hit collection | *Plasmodium* 3D7 asexual blood stage inhibition IC_50_ (nM) | Plasmodium NF54 gametocyte inhibition IC_50_ (nM) | IC_50_ in SMFA (nM) |
| --- | --- | --- | --- | --- |
| MMV1088520 | Malaria | >2000 | 500 | 1078 |
| MMV667494 | Malaria | 7.2 | NT | 18 |
| MMV022029 | Malaria | 800 | 1400 | 56 |
| MMV688122 | Tuberculosis | NT | NT | 1000 |
| MMV675968 | Cryptosporidiosis | NT | NT | 406 |

S4 Table. Compounds analysed in full dose response in the SMFA. *Plasmodium* asexual blood stage and gametocyte inhibition data were retrieved from https://www.mmv.org/mmv-open/pathogen-box
