# Supplementary material for: Barcoded Asaia bacteria enable mosquito in vivo screens and identify novel systemic insecticides and inhibitors of malaria transmission
Source: PLoS Biol. 2021 Dec 20;19(12):e3001426. doi: 10.1371/journal.pbio.3001426 (PMC8726507; doi:10.1371/journal.pbio.3001426)
Supplement: Attachment — Submitted filename: Response to Reviewers.pdf [file pbio.3001426.s015.pdf]

*Reviewer #1: PBIOLOGY-D-21-02346*
*The use of barcoded Asaia bacteria in mosquito in vivo screens for identification of inhibitors of malaria transmission*

*The authors describe a new methodology which can be used to aid the discovery of novel insecticides. Genetically engineered alpha-proteobacteria (Asaia) containing a DNA-barcode was added to bloodmeals in combination with compounds to be tested. A custom-built device was developed to facilitate the feeding protocol. The mosquitoes are pooled according to phenotype and the bacteria grown. The DNA barcodes corresponding to different phenotypes are then amplified and quantified. Barcodes enriched in the dead mosquitoes identified compounds with systemic adulticidal activity. In a subsequent series of experiments this system also allowed for compounds to be tested for their efficacy in targeting sexual stages of the parasite by employing P. falciparum gametocytes expressing a GFP-luciferase fusion protein for detection. By killing the mosquito vector or targeting the sexual stages of the parasite, the transmission of malaria would be interrupted.*

*The manuscript is well written, the methodology is innovative, well explained and might represent a game-changer in screening compounds acting as insecticides or targeting the sexual stages of P. falciparum. The process might even be adapted to other parasitic species which depend on blood-feeding vectors.*

*Comments:*

*Figure 1b - Why was the uptake/fluorescence of barcode B so much lower in some mosquitoes when compared to barcode A?*

The intensity of the fluorescence signal observed in the mosquitoes is the result of sampling of the bloodmeal, amplification of the barcode by growing the Asaia bacteria and PCR amplification of the barcodes, and the efficiency of the Luminex bead in hybridizing to the barcode signal. Variation at all these steps leads to the variation in signal strength seen in Figure 1B. In this experiment we also observed a lower sampling of barcode 2. This may be due to experimental variation and the relatively low sample size in this pilot experiment. Subsequent experiments showed a more even sampling of barcodes as shown in Figures 1C&D and Figure 2B. The experimental variation is mentioned in the results section (lines 126-127). In addition, we have now included a more elaborate discussion of barcode sampling in the discussion section:

"Sampling intensity of *Asaia* encoded bloodmeals differed between wells, and some wells were sampled by more than one mosquito. This is in line with the biting behaviour observed with the Vectorchip that contains arrayed glucose meals to sample saliva from individual mosquito bites (36). By using a surplus of mosquitoes relative to the number of bloodmeals we made sure that every well was sampled by multiple mosquitoes. Small contaminations with barcodes from cross-feeding mosquitoes did not impact overall barcode enrichment in pooled analyses."

*Figure 4D - "The lower left corner of each panel indicates the proportion of barcode signal that was retrieved from uninfected mosquitoes". How was this proportion calculated? Is it a percentage?*

Indeed, this is a percentage and was calculated by:

$$P_u = 100 \times \left(\frac{I_u}{I_u + I_i}\right)$$

Where $I_u$ and $I_i$ are the background corrected median fluorescence intensities in the uninfected and infected mosquitoes, respectively. We have now more explicitly explained this in the materials and methods (lines 526-533).

*"Compounds identified by a blue dot were selected for full dose response analysis". Why were these compounds specifically chosen?*

In the barcoded screening assays and the single point glass membrane feeder assays, compounds were tested at 10 or 20 µM. To gain insight in the range in potencies of the transmission-blocking hit compounds, we randomly selected 5 compounds for full dose response analyses in glass feeder experiments. We have clarified this in the text (lines 259-260).

*Line 235 - Is the number 23 a reference?*

Indeed, this is a reference that was incorrectly placed. It has now been placed at the end of the sentence.

*Line 436 - If plates containing infectious P. falciparum gametocytes of the parasite were incubated for 24h with compounds, would that not generate false positives? The compounds have a 24h window to kill the gametocytes before the addition of the bacteria and before the feeding assays, in an environment which does not reflect their natural habitat.*

We specifically pre-incubated gametocytes with test compounds prior to mosquito feeding in order to identify compounds with a gametocytocidal mode of action. This is in line with the target product profiles references in the introduction section (line 70). We have clarified this in the text in the results section (lines 237-240).
Although indeed artificial, culture conditions for maintenance of infectious *P. falciparum* are well established and our method is in line with these conditions. We have referenced this in the Methods section (lines 483-484).

*Reviewer #2: This is a nicely written paper that cleverly repositions some older ideas into an effective and efficient screen for testing systemic insecticides and Plasmodium transmission blocking (TB) molecules in mosquito blood meal bioassays. This is very novel, important not just for the field but could broadly facilitate discovery of biomolecules that help us control many different mosquito borne diseases, and has already shown effectiveness in drug discovery in the case of the TB assays given the several new effective compounds discovered from the MMV Pathogens Box. Overall, I believe their methods do significantly outperform their predecessors in precision, resolution, speed and accessibility. I think their controls are well done, and prove the efficiency of their tests. I like that they have done a good job of showing that mosquitoes can and do sample multiple wells, but that their barcodes and plate set up allows for discrimination of this and so they can handle*
*the false positives and false negatives that happen from this habit. I think it is very clever that their serial testing adds in lower or higher dose testing of the compounds to verify positive hits by re-testing at a lower dose, and screens for false negatives by re-testing at a higher dose. I think they did their due diligence on their drug hits by going to the standard, repeat membrane feeds using group membrane feeds at serial doses. I have several broad critiques and questions about the present manuscript that I hope the authors can answer.*
*1) The novelty of their idea and technology is reconfiguring some older/other ideas and adding a few new ones to make an efficient screen technology for blood feeding mosquitoes. That said, they should do a better job of describing those foundational ideas, particularly the idea of barcoding mosquitoes/blood meals.*

Indeed the references to these older/other ideas might have been hidden in the original text. We have modified the introduction and indicated that our approach builds on previous work that introduced the concept of large screens using arrays of barcoded bloodmeals (lines 79-85).

*a. The Killeen et al work, buy virtue of using phage display, did not just develop the idea of blood feeding Anopheles on 96 well plates, but also 'bar-coded' the wells by virtue of testing one scFv-displaying phage per well, which contained the genetic sequence of that particular scFv in the phage genome, and phages that had supposed activity were recovered from the guts of dead mosquitoes, re-amplified and re-tested. Ghosh et al (PMID 11687659) did a similar thing in 2001, but did not use the multi-well idea.*

Indeed Killeen et al did conceive the barcoding concept using phages. We have now included a more extensive reference to this work and to the work of Ghosh et al. in the introduction, and indicated that our approach builds on these original ideas:

“Previous work has demonstrated the use of phage display technology to enable large scale screens for peptides that block *Plasmodium* development in the mosquito vector (*24*). Killeen et al. showed the feasibility of feeding mosquitoes on arrays of bloodmeals encoded with scFv-displaying phages that could be recovered, identified, propagated and retested from individual mosquitoes (*25*). This introduced the concept of large-scale screens, where active substances could be identified by enrichment of their cognate phage barcode in mosquitoes with the phenotype of interest. Here we build on this concept and present……”

*b. Bar-coding individual/small groups of mosquitoes for better assay discrimination is also being done a lot now in various ways. For example, it is being used in mark-release-recapture experiments in*

*the field to discriminate different pools of mosquitoes that are released at different times (Faimen et al -*
*c. Ideas like the Vectorchip are testing individual mosquitoes blood feeding on individual wells to discriminate mosquito transmission among a group*.

Thanks for highlighting these interesting preprints that we missed. Both papers have now appeared in peer-reviewed journals and we have included these references and a discussion of the relation to our work in the revised manuscript:

"In capture and release experiments, ectopic DNA oligonucleotides have been used to stably tag mosquitoes during their entire lifespan (*36*). Our data indicate that upon ingestion with the bloodmeal, DNA oligonucleotides are rapidly eliminated from the mosquito in spite of their nuclease-resistant phosphorothioate backbone. In contrast, ingested *Asaia* symbionts stay with the mosquito for life and thereby makes long-term applications possible (26). Sampling intensity of *Asaia* encoded bloodmeals differed between wells, and some wells were sampled by more than one mosquito. This is in line with the biting behaviour observed with the Vectorchip that contains arrayed glucose meals to sample saliva from individual mosquito bites (36). By using a surplus of mosquitoes relative to the number of bloodmeals we made sure that every well was sampled by multiple mosquitoes. Small contaminations with barcodes from cross-feeding mosquitoes did not impact overall barcode enrichment in pooled analyses. In these analyses, the number of PCR reactions needed to detect individual barcodes was significantly reduced by using common amplification regions and multiplex detection of barcode sequences.
"

*2) After reading the whole manuscript, I understood the need to use Asaia for the bar coding - because it colonizes the gut and so allows for detecting the bar code 8 days post blood feeding when the TB assays for oocyst infection needs to be done. However, I was confused early on when the focus was only on systemic mosquito insecticides because I didn't understand why Asaia was needed when the mortality/live screen was only done 2 days post blood meal. To my mind, such a short timeframe would have allowed the investigators to simply put in a genetic barcode oligo or plasmid directly in the wells, and simply do PCR of the guts of the live vs. dead mosquitoes because 2 days is not enough time to digest these in the midgut; adding the barcoded Asaia seems to add an unnecessary culture step that slows the screen. Can the authors explain this? Was the idea just to keep the steps the same between the two types of tests? Would the mortality screen indeed be effective and/go faster if what I described is changed for the mortality screen only?*

When developing the approach we looked for a technology that would allow us to investigate phenotypes that require a longer time to develop, e.g. *Plasmodium* oocyst formation. We initially experimented with DNA oligo nucleotides in the bloodmeal, exactly as the reviewer suggests, but have found that these were very rapidly cleared by the mosquito. We have included these results in the revised version of the paper (Fig. S2A and methods in lines 378-385) to provide a better rationale for the use of *Asaia* to tag the mosquitoes

*3) Similar to the above comment, I am wondering about the use of MagPix for the output of determining bar code relative intensity. Again, it seems it may be needed for the oocyst detections because oocyst number/intensity is the readout, but for a live/dead screen, don't they just need a +/- determination of their assay (PCR or qPCR)…which would make it easier and more efficient because no Asaia culture, nor an expensive MagPix machine and fluorescent beads, is needed? For example, in Fig2 - if the y-axis is a binary determination on whether fipronil (the pos control) or DMSO (the neg control) was sampled, the more complicated bar code relative intensity readout is not really necessary, right?*

The advance of the Magpix is that it allows multiplex analyses. For example, the experiments presented in Figure 2 used 24 distinct barcodes, 12 for the DMSO-containing wells and 12 for the fipronil wells. Thus, each mosquito could contain any of these 24 barcodes or combinations thereof.
Detection of these barcodes in the 147 mosquitoes that were analysed would require 24x147 = 1,764 PCR reactions per replicate. In contrast, using the fluorescent beads and Magpix detection, only 147 PCR reactions were required as the detection of the 24 barcodes could be run in multiplex in a single reaction per PCR product. We have included a sentence in the discussion to highlight the advantage of multiplexing (lines 299-301)

*4) Line 185, they refer to the idea that interference from a gustatory effect was counted out in their testing. This was a good test of screening out artifacts, but actually testing FOR gustatory effects would be interesting and potentially useful in control strategies. Can the authors comment on that?*

This is indeed a very interesting thought. We have included this in a more extensive discussion of other applications of the barcoding method in the discussion section:

"Applications beyond the examples provided in this paper are conceivable. For example, it should be possible to identify gustatory modulators through barcodes not sampled in arrayed screens. The incubation time in the screen for systemic insecticides presented here could be extended to screen for compounds with a slow mode of action, possibly selecting compounds less prone to development of resistance. Since *Asaia* is transmitted vertically, screening for barcodes that are absent on eggs or in progeny may identify mosquito contraceptives that reduce fecundity (*55*)."

*5) Fig 3B shows negative and >100% signals in dead mosquitoes, which is counter-intuitive. I think this is from the idea of multiple wells being sampled by mosquitoes but I'm not sure. Please explain what is going on here.*

The negative and >100% signals originate from the background correction that we applied. All Luminex signals were corrected for background from negative control (buffer only) PCR reactions. In some experiments, the signal from these negative controls was slightly above the signal in negative mosquitoes. As a result, the percentage of signal in the dead mosquitoes could be below zero or above 100. We have now included a more elaborate description of the calculation in the method section as well as an explanation for the negative and >100% values in the result section (lines 199-202).

*6) For the TB effects, the readout is RLU of mosquitoes from oocyst intensity. This is an efficient way to do this to prevent the need to dissect and count oocysts, but it also prevents us from understanding the RLU-to-oocyst number relationship. Please add data that gives us this understanding. Only 1 oocyst is needed to transmit the parasite. If a drug reduces RLU intensity by 50%, it may be statistically technically significant but may be irrelevant in the ability to reduce malaria transmission using the drug (e.g going from 10 down to 5 oocysts is unlikely to affect malaria transmission by the mosquito). This should be acknowledged, and the effectiveness of the positive hits couched in this critique - changing oocyst intensity is only really going to matter if we change it to zero from what ever number.*

This is a very valid point, indeed infection prevalence rather than infection intensity should be reduced in order to impact malaria transmission. In our view, the barcoding technology can serve as a primary screen to identify potential transmission-blocking molecules. The exact efficacy in relation to drug concentration should be investigated in more detailed analyses, like to ones we present in Figure 4E. We have now included the level of background signal in uninfected mosquitoes in Figure 4E to indicate at what compound concentration the infection prevalence is reduced to zero.

We have previously published on the relationship between oocyst intensity and luminescence in mosquitoes infected with luciferase reporter parasites (Stone et al.,2014, PMID 24829466, Vos et al., 2015, PMID 26687564,). We include the relevant figures from these papers here for your reference:

D

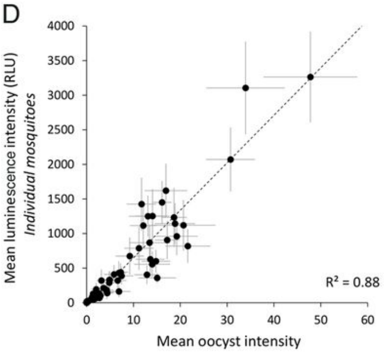


D, Mean oocyst intensity and mean luminescence intensity for 68 feeds (30–58 mosquitoes examined per feed; y = 68.71x – 37.68). In all figures of infection intensity, error bars represent bootstrapped 95% CI.

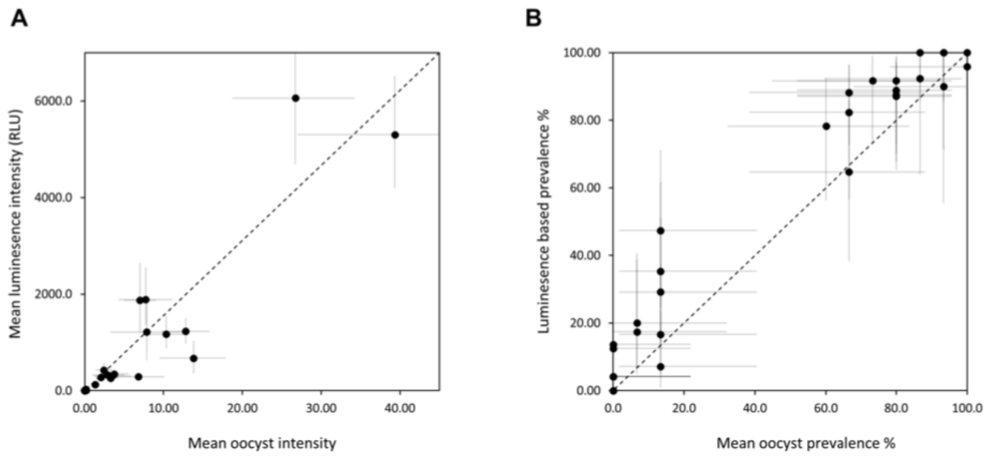


**Figure S3. A.** Relative oocyst and luminescence intensity among mosquitoes sampled from the same cages fed blood-meals without the addition of a test compound (16 feeds, 480 mosquitoes) or with the addition of DHA (16 feeds, 711 mosquitoes). **B.** Relative infection prevalence based on the observation of oocysts or calculated from luminescence intensity, among mosquitoes sampled from the same cages fed blood-meals without the addition of a test compound (16 feeds, 480 mosquitoes) or with the addition of DHA (16 feeds, 711 mosquitoes).

We have clarified in the results section that the luminescence signal is linearly correlated to the number of oocysts in the mosquito midgut with reference to the above papers (lines 228-230).

Furthermore, we modified the discussion to indicate that successful reduction of malaria transmission ultimately depends on the ability to reduce the number of infected mosquitoes, which requires full blockage of parasite development (lines 351-353).

*7) Fig 4e shows a legend for Rep 1 and Rep 2 but with no distinguishment between the two (eg. different colored dots). I wonder if the authors meant to color the individual dots that construct the SMFA dose response analysis by Rep 1 vs. Rep 2?....this would be helpful.*

We used open squares and circles to indicate the difference between the replicated but acknowledge that this was very difficult to see at the light color that was used in the graph. We have now used colors that more clearly indicate the two replicates.

*8) Line 319 - they should briefly explain the rule-of-five principles.*

We have included this in the discussion section (lines 345-347)

*9) Could the authors expound upon the idea that their test could allows for screening of slow killing systemic insecticides, and for screening for egg-laying inhibitors? Both assays would be very useful - the former potentially get hits for chemicals that would potentially be more resistant to insecticide resistance-development and the latter help to reduce the reservoir population.*

Again, these are very interesting thoughts. We've modified the discussion to indicate the potential of the barcoding approach for other types of phenotypic screens, including the ones indicated by the reviewer (lines 358-371). As for screening for egg-laying inhibitors: we have performed a few pilot experiments that confirmed that the Asaia barcodes are vertically transmitted and present on the eggs. However, unambiguous detection of barcodes from egg-laying mothers was disturbed by contaminating barcodes present in mosquito excrements. Successful development of this screening application would require a considerable amount of optimization.

*10) I don't understand what the authors are saying in sentences 392-395. Please revise.*

We meant to say that we made sure that there was an equal number of templates in all PCR amplifications. In a screen, we often saw uneven distribution of phenotypes, e.g. 80 dead and 20 live mosquitoes. When we then wanted to compare pooled barcodes from 80 Asaia cultures from dead mosquitoes to 20 cultures from live mosquitoes, we added 60 culture equivalents of a mock (non-related barcode) to the pool from the live mosquitoes. This to prevent that differences in the number of PCR templates led to differences in amplification efficiencies and a bias in the barcode representation in the PCR products. We have modified the text as follows:

"For comparative analyses (e.g. live versus dead mosquitoes), mock cultures with an unrelated barcode were added to make up for differences in sample sizes between the two pools. This to prevent differences in amplification efficiencies due to different numbers of PCR templates and, as a result, a bias in the barcode representation."

*11) Line 452 - explain "cherry-picked"*

We meant to say that after the analyses of infection status of the mosquito we went back to the Asaia plates and picked all wells corresponding to uninfected mosquitoes and pooled them in a single tube. We did the same for wells corresponding to infected mosquitoes. We have clarified this in the methods section as follows:

"*Asaia* cultures from uninfected and infected mosquitoes were collected in separate pools for further analysis of barcode signals."
